# Model-based contact detection and position control of a fabric soft robot in unknown environments

**DOI:** 10.3389/frobt.2022.997366

**Published:** 2022-10-13

**Authors:** Zhi Qiao, Pham H. Nguyen, Wenlong Zhang

**Affiliations:** ^1^ School for Engineering of Matter Transport and Energy, Ira A. Fulton Schools of Engineering, Arizona State University, Tempe, AZ, United States; ^2^ Aerial Robotics Lab, Imperial College London, London, England, United Kingdom; ^3^ School of Manufacturing Systems and Networks, Ira A. Fulton Schools of Engineering, Arizona State University, Mesa, AZ, United States

**Keywords:** soft robotics, control of soft robots, soft robotics applications, nonlinear disturbance observer, sliding mode control

## Abstract

Soft robots have shown great potential to enable safe interactions with unknown environments due to their inherent compliance and variable stiffness. However, without knowledge of potential contacts, a soft robot could exhibit rigid behaviors in a goal-reaching task and collide into obstacles. In this paper, we introduce a Sliding Mode Augmented by Reactive Transitioning (SMART) controller to detect the contact events, adjust the robot’s desired trajectory, and reject estimated disturbances in a goal reaching task. We employ a sliding mode controller to track the desired trajectory with a nonlinear disturbance observer (NDOB) to estimate the lumped disturbance, and a switching algorithm to adjust the desired robot trajectories. The proposed controller is validated on a pneumatic-driven fabric soft robot whose dynamics is described by a new extended rigid-arm model to fit the actuator design. A stability analysis of the proposed controller is also presented. Experimental results show that, despite modeling uncertainties, the robot can detect obstacles, adjust the reference trajectories to maintain compliance, and recover to track the original desired path once the obstacle is removed. Without force sensors, the proposed model-based controller can adjust the robot’s stiffness based on the estimated disturbance to achieve goal reaching and compliant interaction with unknown obstacles.

## 1 Introduction

There has been a growing interest in using pneumatic actuators and fabric materials to design soft robots over the recent years Nguyen et al. (2019); Best et al. (2016); Hofer and D’Andrea (2020). Pneumatic-actuated fabric-based robots have shown desirable characteristics such as being lightweight, customizable, robust, and highly compliant. By changing the internal pressure, these fabric-based soft robots can vary their stiffness from almost zero to a substantial value. Both linear and nonlinear model-based controllers have been designed to drive these robots to their desired trajectories, such as iterative learning control Hofer and D’Andrea (2020), model predictive control and sliding mode control Best et al. (2021), robust backstepping control Wang et al. (2019), and adaptive control Trumić et al. (2021), just name a few. Different designs of model-based controllers and their associated challenges are detailed in a recent review paper Della Santina et al. (2021). However, both linear and nonlinear control approaches need to increase the soft robot’s stiffness to be responsive to tracking errors. This increased stiffness requirement makes the soft robot behaves more like its rigid counterparts. Once the soft robot reaches a high stiffness value, it could also lead to hard collisions and loss of safety when humans and unknown obstacles are on its path. However, it is still an open challenge for a soft robot to detect possible contacts with the environment, stay compliant with obstacles, and adjust the robot’s path if needed.

For soft robots, both body-integrated soft sensors and observer-based approaches have been explored to detect contact events. Based on the existence and properties of the obstacles, a soft robot can switch between different control strategies. Capacitive and resistive sensors are commonly integrated with soft robots. Capacitive sensors Frutiger et al. (2015); Totaro et al. (2020); Navarro et al. (2020) are compliant, but they have a proximity effect to conductive objects, limiting their real-world application. Resistive sensors Elgeneidy et al. (2018); Thuruthel et al. (2019); Rosle et al. (2022) often exhibit nonlinear time-variant behaviors, making it challenging to calibrate and model them. Moreover, although body-integrated soft sensors provide direct contact force estimation Wang et al. (2018), they need to be placed in the contact area of the robot body, which may be unknown when the robot operates in cluttered environments. In contrast, observer-based approaches leverage dynamic models of the robots for contact force/torque estimation using more accessible input/output measurements. In Bui and Schultz (2021), a disc-thread-based linear parameter varying model was proposed for a fabric-reinforced actuator. Each disc was modeled as a sub-system, and a third-order sliding mode observer was created for each sub-system to estimate the contact force. However, the computational cost increases with the growing number of discs used in the model, and applying this method to large-scale robots is still challenging. In Navarro et al. (2020), both force intensity and deformation were estimated by integrating a FEM-based numerical model with capacitive and pneumatic sensor feedback. The capacitive sensor estimated the contact location, while the pneumatic sensor quantified the deformation. The FEM model mediated measurements from both sensors to estimate the contact force and the robot’s deformation. However, finding the FEM model for the fabric-based soft robot is difficult due to the high uncertainties and compliance.

In this article, we present a Sliding Mode Augmented by Reactive Transitioning (SMART) controller, which switches the position reference for the baseline sliding mode controller (SMC) based on the contact events detected by a nonlinear disturbance observer (NDOB). The NDOB utilizes the kinematic and pressure measurements to estimate the lumped disturbance, including modeling uncertainties and unknown external torque applied on the actuator’s tip during contact with the environment. The switching algorithm utilizes the tracking error and estimated disturbance to detect the contact and accordingly switch among three modes (tracking, compliance, and recovery). We proved that the proposed controller ensures that the tracking error between the current position and the switched position reference is ultimately bounded under the effect of the lumped disturbance. Experimental results show that the SMART controller can successfully detect obstacles, become compliant at its current position, and return to the desired trajectory when the obstacle is removed, even with the presence of modeling uncertainties.

This article is inspired by the work in Santina et al. (2020), where a machine learning-based disturbance observer was designed to estimate the contact force between a silicone-based soft arm and the external environment. In Santina et al. (2020), a piecewise constant curvature (PCC) model, assuming the existence of a backbone, was utilized to describe the dynamics of the silicone-based soft arm, and the modeling errors were learned from experimental data through Gaussian process regression. In this article, a new parameter is introduced to extend the PCC model to fabric-based actuators with a design of a hollow center and no backbone. Instead of utilizing a machine learning approach to compensate for the modeling uncertainties, those uncertainties are handled successfully by a model-based NDOB and SMC. The training data set is not required for the proposed method compared with the machine learning approach. In addition, the proposed method also closes the position control loop using the estimation results.

In summary, this work contributes to.

• A novel dynamic model for a hollow-centered fabric actuator with parameter identification and model validation.
• An NDOB that estimates the lumped disturbance, including model uncertainties and contact torque.
• A SMART controller utilizes the estimated disturbance to adjust the desired trajectory with the ultimate boundness proof of the tracking error.

The remainder of the paper is organized as follows: Section 2 introduces the design and dynamic model of the fabric-based robot. Section 3 presents the design of the baseline SMC approach and the proposed SMART controller. Section 4 presents the hardware setup and the evaluation experiments’ design. Section 5 presents the evaluation results of the dynamic model accuracy, the performance comparison between the baseline SMC and the proposed approaches, and the evaluation of the parameter for the SMART controller design. Section 6 concludes this paper and discusses future directions.

## 2 Actuator design and dynamic model

### 2.1 Design of the fabric soft robot

**FIGURE 1:**
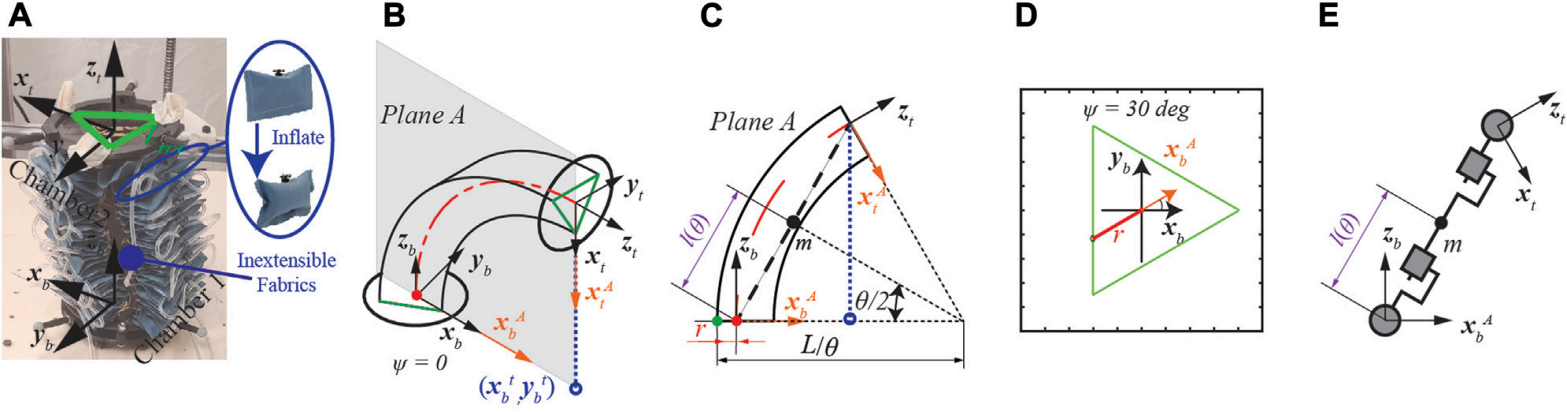
Modified augmented rigid arm model for the fabric-based actuator. **(A)** Isometric view of the actuator. **(B)** Curvature plane with rotation angle ψ = 0deg. **(C)** The modified model with the addition of parameter r on the curvature plane. The green and red lines indicate the inextensible layer and the axial line, respectively. **(D)** Top view of bottom plate when ψ = 30deg. **(E)** Augmented rigid arm (RPPR).

The fabric-based soft robot used in this paper was introduced in our previous work Nguyen et al. (2019).In a single segment, the robot contains two parts: rectangular-shaped air pillows and an inextensible fabric layer with three lines of pouches, as presented in Figure 1A. Each pouch holds one air pillow, and three individual chambers are created by serially connecting all the pillows on the same line. When the actuator is inflated, the pillows in the pouch push each other and create a bending motion. With three segments, the soft robot can lift a load ten times heavier than its body weight (1.1 kg) by wrapping around the object, and it can also be compressed to half of its original length when the chambers are not inflated. However, material compliance, fabrication errors, and inconsistent interaction between pillows introduce significant actuator modeling and control challenges.

### 2.2 Dynamic modeling of fabric bending actuators

The augmented rigid-arm model for soft robotics was first proposed for an elastomeric-based actuator in Della Santina et al. (2018). The proposed approach follows the PCC assumption with the inextensible arc (also known as the virtual backbone) located at the axial line of the actuator Marchese et al. (2015). However, the inextensible arc for our fabric-based robot is designed in a triangular shape, shown in Figure 1A. When inflated to different pressure values, the inextensible arc will move along the edge of the triangle. And the offset between the axial line and the inextensible layer of the actuator will make the existing models inaccurate.

As a preliminary study, only one segment is utilized in this work. As shown in Figure 1A, axis **z** is perpendicular to its local plate, and axis **x** positive direction goes through the connection point between chambers 2 and 3 on its local plate. The orientation of the actuator is described by two parameters $\psi \in \left[0,2\pi \right)$ and $\theta \in \left[-\frac{\pi }{2},+\frac{\pi }{2}\right]$ , ψ represents the counterclockwise rotation angle between the plane **z**_b_ − **x**_b_ and the PlaneA where the curvature happens, as shown in Figure 1B. θ represents the angle between the reference frame $x_{b}^{A}$ and **x**_t_ expressed on the curvature plane, presented in Figure 1C. Since the actuator was designed to reach a high elevation angle and the rotation motion on the azimuth direction was less regulated, we only control the pressure in chamber 1 and focus on tracking the bending angle θ of in this paper.

To match the actuator design, we introduce a new parameter r into the augmented robot model, as shown in Figures 1C,D. Solid green dot is the intersection point between the constant curvature arc and the bottom plate while solid red dot is the origin of Plane A. r represents the distance between the aforementioned green and read dot and is calculated as follows:

$$\begin{array}{ll} \psi & =atan\left(\frac{y_{b}^{t}}{x_{b}^{t}}\right),\\ r & =\frac{l_{\mathrm{tri}}}{\sqrt{3}}\frac{\cos\left(\pi /3\right)}{\cos\left(\left(\left(\psi +\pi \right)\;\mathrm{mod}\;2\pi /3\right)-\pi /3\right)}. \end{array}$$ (1)

Where point $\left(x_{b}^{t},y_{b}^{t}\right)$ is the projection of the top plate’s center point on the bottom plate, l_tri_ is the length of the edge of the equilateral triangle and marked as the green line in Figures 1A,D. Eq.1 is originated from a base function: $r=\frac{\cos\left(\pi /3\right)}{\cos\left(\psi -\pi /3\right)}$ . The base function represents a line goes through two points: (1, 0) and (cos (2π/3), sin (2π/3)). If ψ is restricted within [0, 2π/3], the drawing of one edge of the triangle shape is complete. The modulus function ψ mod  2π/3 is used to draw the other two edges and complete the triangle shape. $\frac{l_{\mathrm{tri}}}{\sqrt{3}}$ enlarge the unit triangle to match the actuator’s design while changing the denominator to cos (((ψ + π) mod  2π/3) − π/3) rotates the triangle shape to match coordinate of the soft arm.

The Denavit-Hartenberg (DH) parameters for the augmented rigid robot are presented in Table 1. Two prismatic joints and two revolutionary joints (RPPR) are used to describe the dynamics of the augmented rigid robot, as shown in Figure 1E. The configuration is described by joint space vector $\Gamma ={\left[\frac{\theta }{2},l\left(\theta \right),l\left(\theta \right),\frac{\theta }{2}\right]}^{T}$ where l(θ) is calculated as follows:

$$l\left(\theta \right)=\left(\frac{L}{\theta }-\mathrm{sign}\left(\theta \right)r\right)\sin\left(\frac{\theta }{2}\right).$$ (2)

**TABLE 1:** Classic DH parameters θ, d, a, α and mass m for one actuator.

Link	Θ	d	a	α	m
1	$\frac{\theta }{2}$	0	0	$-\frac{\pi }{2}$	0
2	0	l(θ)	0	0	m_i_
3	0	l(θ)	0	$\frac{\pi }{2}$	0
4	$\frac{\theta }{2}$	0	0	$-\frac{\pi }{2}$	0

Following the derivation presented in Della Santina et al. (2018), the partial derivative of the joint space vector Γ with respect to the bending variable θ is presented as

$$J_{\Gamma }\left(\theta \right)=\frac{\partial \Gamma }{\partial \theta }={\left[\frac{1}{2},h\left(\theta \right),h\left(\theta \right),\frac{1}{2}\right]}^{T}.$$ (3)

where $h\left(\theta \right)=\frac{1}{2}\left(\frac{L}{\theta }-r\right)\cos\left(\frac{\theta }{2}\right)-\frac{L}{\theta^{2}}\sin\left(\frac{\theta }{2}\right)$ .

We introduce the stiffness and damping terms to complete the dynamic model of the soft arm as:

$$M\left(\theta \right)\ddot{\theta }+C\left(\theta ,\dot{\theta }\right)\dot{\theta }+G\left(\theta \right)+K\theta +D\dot{\theta }=\alpha R\left(\psi \right)p+\tau_{\mathrm{ext}}$$ (4)

Where

$$\begin{array}{ll} M\left(\theta \right) & =m\left(\frac{r^{2}}{4}+{\left(\frac{\cos\left(\frac{\theta }{2}\right)\left(r-\frac{L}{\theta }\right)}{2}+\frac{L\sin\left(\frac{\theta }{2}\right)}{\theta^{2}}\right)}^{2}+\frac{\sin{\left(\frac{\theta }{2}\right)}^{2}{\left(r-\frac{L}{\theta }\right)}^{2}}{4}\right),\\ C\left(\theta ,\dot{\theta }\right) & =-m\left(\right.\left(\frac{\cos\left(\frac{\theta }{2}\right)\left(r-\frac{L}{\theta }\right)}{2}+\frac{L\sin\left(\frac{\theta }{2}\right)}{\theta^{2}}\right)\left(\frac{\dot{\theta }\sin\left(\frac{\theta }{2}\right)\left(r-\frac{L}{\theta }\right)}{4}-\frac{L\dot{\theta }\cos\left(\frac{\theta }{2}\right)}{\theta^{2}}+\frac{2L\dot{\theta }\sin\left(\frac{\theta }{2}\right)}{\theta^{3}}\right)\\ & \;+\frac{\dot{\theta }\sin\left(\frac{\theta }{2}\right)\left(r-\frac{L}{\theta }\right)\left(\frac{\cos\left(\frac{\theta }{2}\right)\left(r-\frac{L}{\theta }\right)}{2}+\frac{L\sin\left(\frac{\theta }{2}\right)}{\theta^{2}}\right)}{4}\left.\right),\\ G\left(\theta \right) & =-mg\frac{L\sin\left(\theta \right)+r\theta^{2}\cos\left(\theta \right)-L\theta \cos\left(\theta \right)}{2\theta^{2}},\\ \tau_{\mathrm{ext}} & =J\left(\theta \right)f_{\mathrm{ext}},\\ R\left(\psi \right) & =\left[\sin\psi \;-\cos\psi \right]\left[\begin{array}{ccc} -\sin\left(\frac{\pi }{6}\right) & \;-\sin\left(\frac{\pi }{6}\right) & 1\\ \cos\left(\frac{\pi }{6}\right) & \;-\cos\left(\frac{\pi }{6}\right) & 0 \end{array}\right]. \end{array}$$

M, C, and G are the inertia term, centrifugal and Coriolis term, and gravity term, respectively. J^T^(q) maps the unknown external wrenches f_ext_ to the actuator’s joint torque while R(ψ) maps the pneumatic forces to the joint torque on the bending direction. $p={\left[p_{m_{1}},p_{m_{2}},p_{m_{3}}\right]}^{T}$ is the chamber pressure vector. Stiffness, damping and amplifying coefficients K, D and α are experimentally identified and detailed in Section 5.

## 3 Design of the sliding mode augmented by reactive transitioning controller

In this article, we will design a nonlinear disturbance observer (NDOB) to estimate the lumped disturbance in real-time. We also focus on slowly changing disturbances (Δ) between the actuator tip and the environment. Therefore, we assume $|\dot{\Delta }|\leq \gamma$ , where γ denotes the upper bound of the time derivative of the disturbance. The conventional algorithm that only integrates SMC with NDOB is designed to reject the lumped disturbance entirely. Such rejection also leads to a high actuator stiffness during contact with an obstacle. To resolve this issue, we introduce a three-mode switching algorithm to detect the contact events and adjust the robot’s desired trajectory if needed.

### 3.1 Design of the baseline SMC

Considering the modeling uncertainties, we rewrite Eq. (4) as:

$$\ddot{\theta }=f\left(\theta ,\dot{\theta }\right)+b\left(\theta \right)u+\Delta ,$$ (5)

where

$$\begin{array}{ll} f\left(\theta ,\dot{\theta }\right) & =-M{\left(\theta \right)}^{-1}\left[\left(C\left(\theta ,\dot{\theta }\right)+d_{0}\right)\dot{\theta }+k_{0}\theta +G\left(\theta \right)\right],\\ b\left(\theta \right) & =\frac{\alpha_{0}}{M\left(\theta \right)},\\ \Delta & =-\Delta k\frac{\theta }{M\left(\theta \right)}-\Delta d\frac{\theta }{M\left(\theta \right)}+\Delta \alpha \frac{\alpha_{0}}{M\left(\theta \right)}+\tau_{\mathrm{ext}},\\ u & =R\left(\psi \right)p. \end{array}$$

|Δ*k*| < *K*_*m*_, |Δ*d*| < *D*_*m*_, |Δ*α*| < α_*m*_, and |*τ*_*ext*_| < *τ*_*m*_. Δ*k*, Δ*d*, Δ*α*, and *τ*_*ext*_ denote the parameter uncertainties and unknown disturbance torque while *K*_*m*_, *D*_*m*_, *α*_*m*_, and *τ*_*m*_ represent their upper bounds.

The tracking error, the sliding surface, and the derivative of the sliding surface are defined as

$$\begin{array}{ll} e & =\theta -\theta_{d},\\ \sigma & =\dot{e}+\lambda e,\;\lambda >0,\\ \dot{\sigma } & =\lambda \dot{e}-{\ddot{\theta }}_{d}+f\left(\theta ,\dot{\theta }\right)+b\left(\theta \right)u+\Delta . \end{array}$$ (6)

With the reaching law $\dot{\sigma }=-\eta \mathrm{sign}\left(\sigma \right)$ , the control input is designed as

$$u=b^{-1}\left(\theta \right)\left({\ddot{\theta }}_{d}-\lambda \dot{e}-f\left(\theta ,\dot{\theta }\right)-\eta \mathrm{sign}\left(\sigma \right)\right).$$ (7)

where

$$\eta \geq \frac{1}{1+\alpha_{m}}\left(\alpha_{m}|\lambda \dot{e}-{\ddot{\theta }}_{d}+f\left(\theta ,\dot{\theta }\right)|,+|\frac{K_{m}\theta }{M\left(\theta \right)}|+|\frac{D_{m}\dot{\theta }}{M\left(\theta \right)}|+|\frac{\tau_{m}}{M\left(\theta \right)}|+\epsilon \right).$$

ϵ is a positive number. A larger η drives the system to the sliding surface faster with more significant chattering in the control input. The stability proof for the resultant closed-loop system is straightforward and thus omitted here.

### 3.2 SMC with disturbance estimation

A modified disturbance observer is designed as follows Chen (2003).

$$\hat{\Delta }=\hat{z}+w\left(\sigma \right).$$ (8)

Where $\hat{z}$ and w(σ) are the internal state and the customized function to be designed for the NDOB, $\hat{\Delta }$ is the estimate of the lumped disturbance. In this paper, we choose w(σ) = k_w_σ, k_w_ > 0.

Differentiating (8) and plugging in (6) yields

$$\dot{\hat{\Delta }}=\dot{\hat{z}}+k_{w}\left(\lambda \dot{e}-{\ddot{\theta }}_{d}+f\left(\theta ,\dot{\theta }\right)+b\left(\theta \right)u+\Delta \right).$$ (9)

The control input u is designed as

$$u=u_{eq}+u_{s}+u_{n}.$$ (10)

where

$$\begin{array}{ll} u_{eq} & =-b{\left(\theta \right)}^{-1}\left(f\left(\theta ,\dot{\theta }\right)+\lambda \dot{e}-\ddot{x_{d}}+k_{sf}\sigma \right),\\ u_{n} & =-b{\left(\theta \right)}^{-1}\hat{\Delta },\\ u_{s} & =-b{\left(\theta \right)}^{-1}\eta sat\left(\sigma \right),\\ sat\left(\sigma \right) & =\left\{\begin{array}{cc} \mathrm{sign}\left(\sigma \right)\; & |\sigma |>\mu \\ \frac{\sigma }{\mu }\; & |\sigma |\leq \mu \end{array}\right. \end{array}$$

u_eq_ cancels the known dynamics, u_n_ rejects the estimated unknown dynamics, and u_s_ drives the system to the sliding surface, respectively. sat(σ) is a saturation function to replace the sign(σ) function to mitigate the chattering problem. k_sf_, η, λ and μ are positive constants and selected by the user. redThe results of changing the control parameter’s value are also presented in Section 5.3.

Plugging in control input (10) into (6) yields the following closed-loop dynamics of the sliding surface

$$\dot{\sigma }=-k_{sf}\sigma -\eta sat\left(\sigma \right)+\tilde{\Delta }.$$ (11)

where $\tilde{\Delta }=\Delta -\hat{\Delta }$ . It indicates the system tracking error will reach zero if the disturbance estimation error $\tilde{\Delta }$ reaches zero.

Combining (10) and (9) leads to the following observer dynamics

$$\dot{\hat{\Delta }}=\dot{\hat{z}}+k_{w}\left(-k_{sf}\sigma +b\left(\theta \right)\left(u_{n}+u_{s}\right)+\Delta \right).$$ (12)

The update law for $\dot{\hat{z}}$ is selected as:

$$\dot{\hat{z}}=-k_{w}\left(-k_{sf}\sigma +b\left(\theta \right)\left(u_{n}+u_{s}\right)+\hat{\Delta }\right).$$ (13)

As a result, the closed-loop disturbance estimate and estimation error dynamics are derived by plugging (13) into (12)

$$\begin{array}{ll} \dot{\hat{\Delta }} & =k_{w}\tilde{\Delta },\\ \dot{\tilde{\Delta }} & =-k_{w}\tilde{\Delta }+\dot{\Delta }. \end{array}$$ (14)

This suggests that the estimation error $\tilde{\Delta }$ is bounded, given the upper bound for the rate of disturbance.

Theorem 1. Consider the closed loop system of (5),(8) and (10) under the assumption that $|\dot{\Delta }|\leq \gamma$ . The dynamics of σ and estimation error $\tilde{\Delta }$ is ultimately bounded.

Proof. The Lyapunov candidate function is:

$$V=\frac{1}{2}\sigma^{2}+\frac{1}{2}{\tilde{\Delta }}^{2}.$$ (15)

Taking the time derivative and plugging in (11) and 14 yields

$$\dot{V}=-k_{sf}\sigma^{2}-\eta sat\left(\sigma \right)\sigma +\tilde{\Delta }\sigma -k_{w}{\tilde{\Delta }}^{2}+\tilde{\Delta }\dot{\Delta }.$$ (16)

Using Young’s inequality in Trench (2013) yields

$$\begin{array}{ll} \sigma \tilde{\Delta } & \leq 0.5\left(\sigma^{2}+{\tilde{\Delta }}^{2}\right),\\ \dot{V} & \leq -\left(k_{sf}-0.5\right)\sigma^{2}-\eta sat\left(\sigma \right)\sigma -\left(k_{w}-0.5\right){\tilde{\Delta }}^{2}+\gamma |\tilde{\Delta }|,\\ & =|\sigma |\left(-\left(k_{sf}-0.5\right)-\eta |\sigma |\right)+|\tilde{\Delta }|\left(-\left(k_{w}-0.5\right)|\tilde{\Delta }|+\gamma \right). \end{array}$$ (17)

According to Theorem in Corless and Leitmann (1981), the dynamics of σ and estimation error $\tilde{\Delta }$ is ultimately bounded and these two terms can be enforced into arbitrarily small regions. The bounds on $\left|\tilde{\Delta }\right|$ and |σ| are.

$$|\tilde{\Delta }|\leq \beta =\frac{\gamma }{k_{w}-0.5}$$ (18)

$$|\sigma |\leq \left\{\begin{array}{cc} \frac{\beta -\eta }{k_{sf}}\; & |\sigma |>\mu \\ \frac{\beta \mu }{k_{sf}\mu +\eta }\; & |\sigma |\leq \mu \end{array}\right.$$ (19)

### 3.3 Switching algorithm

**FIGURE 2:**
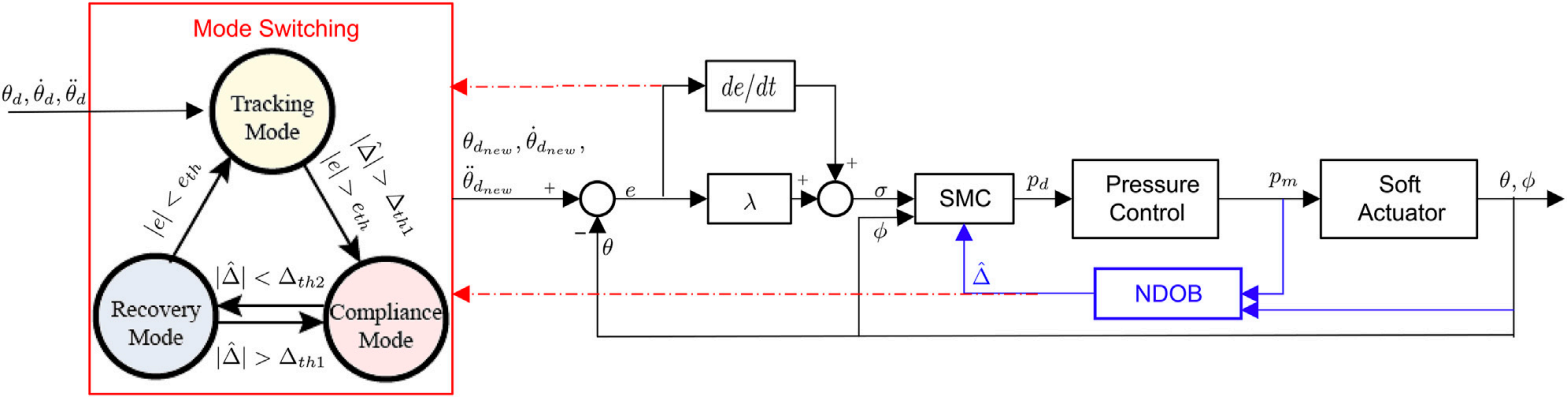
Block diagrams of the SMART controller, which consists of a robust baseline controller (SMC), a disturbance estimator (NDOB), and mode switching algorithm based on the estimated disturbance and position feedback.

A three-mode switching algorithm is introduced to the SMART controller to detect the contact events and adjust the robot’s desired trajectory if needed, as presented in Figure 2. The three modes are defined as tracking, compliance, and recovery. With the thresholds of tracking error measurement (e_th_) and estimated disturbance (Δ_th1_ and Δ_th2_), the desired trajectory switches between the actuator’s original trajectory, actuator’s current bending angle, and a ramp trajectory from the current bending angle to the current set-point, respectively.

We assume that the robot is not initially in contact with the obstacle, so it is in tracking mode. The controller switches from tracking to compliance mode when the magnitudes of the estimated lumped disturbance and tracking error both go beyond the thresholds (Δ_th1_ and e_th_). The estimated lumped disturbance reduces accordingly by adjusting the reference from its original value to the current bending angle, so the actuator will not be further pressurized to squeeze the obstacle. When the estimated disturbance drops below the threshold (Δ_th2_) in the compliance mode, it indicates that the actuator may no longer be in contact with the obstacle, so the controller will switch to the recovery mode to reach the current set-point. The slope of the ramp signal is chosen as 2deg /s to avoid aggressive movement after the obstacle is removed. When the lumped disturbance is above the threshold (Δ_th1_) again in the recovery mode, the controller will switch back to the compliance mode. Otherwise, the controller stays in the recovery mode until the tracking errors are below the threshold (e_th_) and return to tracking mode. The thresholds in the switching algorithm can be tuned to adjust the actuator’s behaviors. Larger values of eth and Δ_th1_ indicate that the controller is less sensitive to the possible contact events. We identify the lower bound of Δ_th1_ when there is no obstacle in the original trajectory so that the modeling uncertainties will not trigger the false detection.

It should be noted that the adjustment of reference position in compliance mode means that the robot waits for the obstacle to be moved out of the original trajectory. The switching algorithm can be easily extended so that the soft robot can explore motions in other directions to move around the obstacles. Therefore, autonomous trajectory re-planning and contact-based navigation Lu et al. (2021); Dicker et al. (2018) is not the focus of this paper; instead, we demonstrate that the soft robot, despite modeling uncertainties, can use a model-based robust control and disturbance observer to detect contact and adaptively reject disturbance. Therefore, this letter proposes a simple trajectory adjustment strategy and evaluates simple trajectory adjustment strategy.

## 4 Experimental setup

### 4.1 System setup

Two Raspberry Pi 3B were utilized for the system’s high-level and low-level control loops. The high-level control loop reads the position feedback from the motion capture system (Optitrack, NaturalPoint, Inc., Corvallis, OR) with six cameras sampling at 120Hz and generating desired pressure profiles at 100Hz. Once the pressure profile is received from the high-level controller, the low-level loop utilizes three pressure regulators (ITV1050, SMC Corporation, Tokyo, Japan) to adjust the air pressure within each chamber. Air pressure is also measured by those regulators and sent back to the high-level controller. The low-level loop also runs at 100Hz.

### 4.2 Model validation and identification

#### 4.2.1 Model validation

In order to validate the proposed augmented model’s accuracy, the actuator was excited with a ramp signal ranging from 0.006 MPa to 0.275 MPa. The bounds of the input signal were selected to ensure they were within the actuator’s operational range. The total duration for each trial was 60 s, with three trials in total. The experimental data and simulation results are compared to validate the proposed augmented model’s accuracy.

### 4.2.2 Identification of parameter uncertainties

In order to identify the bounds of modeling uncertainties, the actuator was excited with a sum of sinusoidal signals as follows:

$$u_{pd}=\overset{10}{\underset{j=1}{\sum }}Amp*\sin\left(2\pi f_{j}t+\phi_{j}\right)+b_{\mathrm{off}}.$$ (20)

where Amp = 1.25 and b_off_ = 1.25 were selected to ensure input u_pd_ stayed within the operational range. Ten different frequency components f_j_ were uniformly selected from [0.001Hz, 0.1Hz] and phase constant ϕ_j_ ∈ [0, 2π] was randomly selected. The time interval for each trial was 60 s, and nine trials were collected in total. The experimental data is utilized to identify the bounds of modeling uncertainties.

### 4.3 Comparison of controller performance

Three sets of experiments were designed to compare the performance of the SMART controller and the benchmark controller (SMC). In all three sets of experiments, all the chambers were pre-inflated to 0.006 MPa, and we only controlled the pressure of chamber 1 to follow a trapezoid bending angle profile. In the first set of experiments, no obstacle was placed in the middle of the robot’s desired trajectory. The goal of the first experiment was to evaluate the bending angle tracking performance. A stiff obstacle (wood plate) was first placed at the marked position in the second set. After 22 s from the start of the trial, the obstacle was moved out permanently. The move-out timing was selected such that the robot was moving to the goal location. An alarm was also utilized to ensure consistency across all the trials. In the third experiment, a compliant obstacle (green grape) was fixed at another marked position, utilizing a trapezoid trajectory with a shorter duration at the flat zone.

### 4.4 Evaluation of the SMART controller design parameters

Four sets of experiments were designed to investigate how k_w_, k_sf_, η and λ impact the system performance. The experiments for k_w_, k_sf_ are similar to the third experiment in the previous subsection, where a 3D-printed stiff obstacle was fixed permanently. A trapezoid bending angle profile was assigned to the actuator. For each parameter, three different values (k_sf_ = 10, 50, 100 and k_w_ = 1, 5, 20) were selected. For η and λ parameters (η = 0.1, 10, 100 and λ = 0.1, 10, 100), a trapezoid bending angle profile was also assigned to the actuator, but no obstacle was used.

## 5 Experimental results

### 5.1 Model validation and identification

#### 5.1.1 Model validation

**FIGURE 3:**
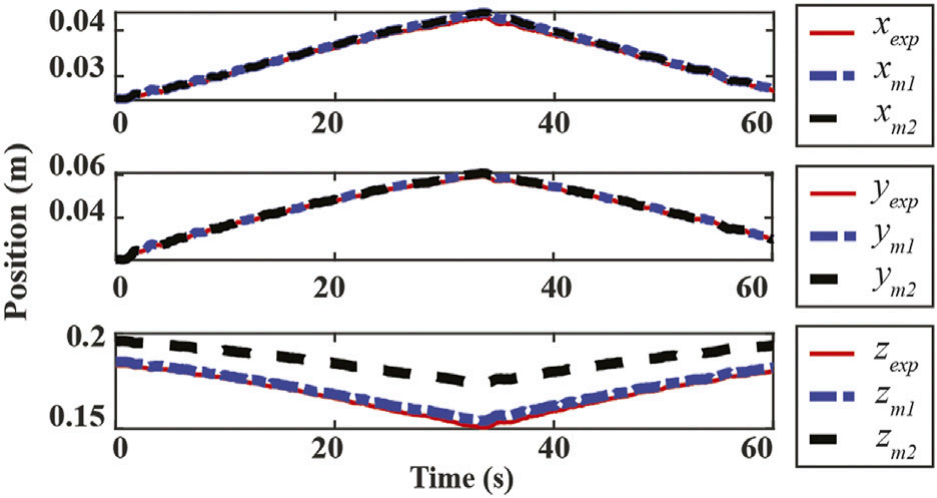
Experimental results for the proposed augmented model. x_exp_, y_exp_ and z_exp_ are the motion capture measurements, respectively. x_m1_, y_m1_ and z_m1_ are the proposed model prediction results. x_m2_, y_m2_ and z_m2_ are the conventional PCC model (without r parameter) results.

The result from one trial is presented in Figure 3 to compare the forward kinematics prediction to the motion capture measurement of the center point of the top plate. In the presented trial, the proposed augmented model is shown to be more accurate posture prediction than the conventional PCC model in the **z**-direction, and the prediction error was found to be less than 3% normalized error in all ranges of motion.

### 5.1.2 Identification of parameters uncertainties

**FIGURE 4:**
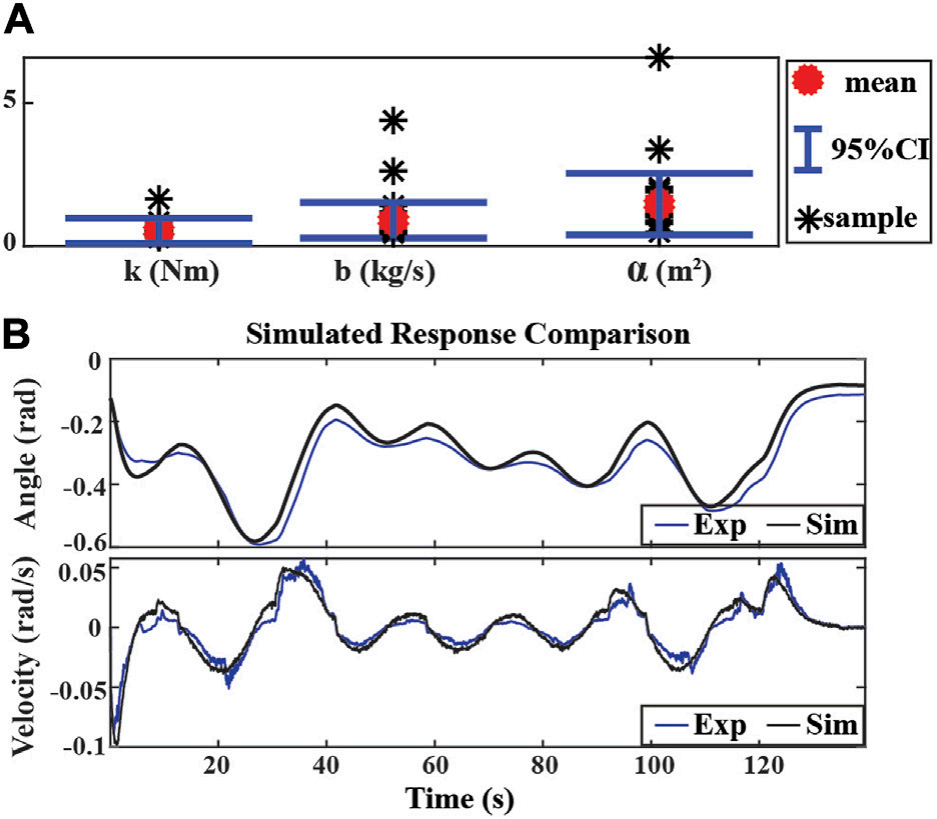
System identification results and simulation. **(A)** Boundaries of parameter uncertainties with nine trials of experimental data. Mean values: k_0_ = 0.4897, d_0_ = 0.8616, α_0_ = 1.2634, standard deviations:Δk = 0.4345, Δd = 0.6600, Δα = 1.0183. **(B)** Simulated response comparison of experimental result (solid blue) and simulation result (solid black).

All parameters were identified using the MATLAB grey-box estimation toolbox with the lsqnonlin algorithm for each trial. The mean value of all the trials is calculated and used as the identified model (k_0_(Nm), d_0_(kg/s), and α_0_(m^2^)). The 95% confident interval of the mean value was taken as the bounds of the modeling uncertainties (Δk, Δd, and Δα). The results are presented in Figure 4A where k_0_ = 0.4897, d_0_ = 0.8616, α_0_ = 1.2634, and Δk = 0.4345, Δd = 0.6600, Δα = 1.0183. The simulation result of the model with mean parameter values is also compared with the experimental data as shown in Figure 4B. It is noted that the model with mean parameter values can capture the system dynamics reasonably well, but noticeable modeling uncertainties remain in the system model.

### 5.2 Comparison of controller performance

**FIGURE 5:**
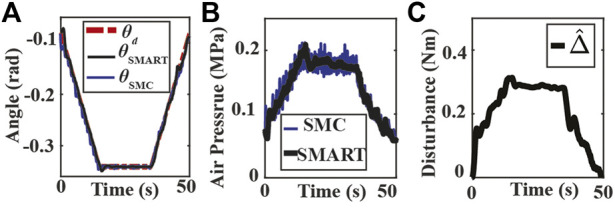
Experiment results of bending angle tracking without an obstacle. **(A)** Tracking results using the SMART and SMC approaches where θ_d_, θ_SMART_ and θ_SMC_ are the desired (dash-dotted red) and the measured bending angles of SMART approach (solid black) and SMC approach (solid blue). **(B)** Measured chamber pressures from SMC (solid blue) and SMART (solid black) controller experiments. **(C)** Estimated lumped disturbance.

The experimental results for the first scenario are in Figure 5. The angular position tracking performance for the SMC and the SMART approach is presented in Figure 5A, where both controllers can track the trapezoid signal. Since the SMC controller rejects the lumped disturbances, including input signal uncertainties, it presented more chattering in chamber pressure measurement, presented in Figure 5B. The reduced oscillation of chamber pressure measurements is also observed for the SMART controller since the NDOB in the proposed controller can adaptively reject the disturbance. The estimated lumped disturbance during position tracking is presented in Figure 5C, where the maximum value of the estimated lumped disturbance is 0.31Nm. To avoid false contact detection, Δ_th1_ = 0.5 is selected for the proposed SMART controller.

**FIGURE 6:**
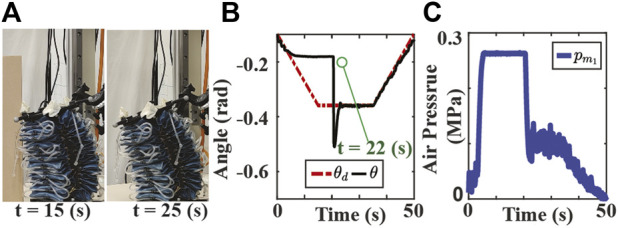
Experiment results of SMC approach tracking bending angle with stiff obstacle where the obstacle is removed permanently at 22 s. **(A)** The screen shots of the experiment video. **(B)** Bending angle tracking results where θ and θ_d_ are the measured (solid black), and the original desired (dash-dotted red) bending angles, respectively. **(C)** Air pressure measurements of chamber 1.

**FIGURE 7:**
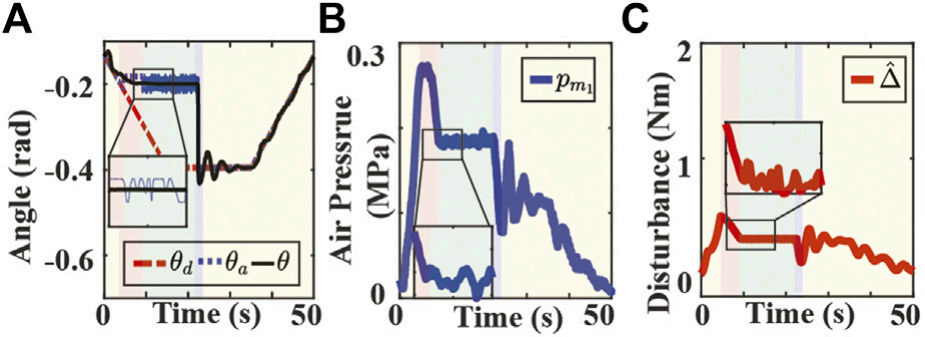
Experimental results of the SMART approach tracking bending angle with stiff obstacle. **(A)** Bending angle tracking results where θ, θ_d_, θ_a_ are the measured (solid black), the original desired (dash-dotted red), and the adjusted desired (dotted blue) bending angles, respectively. **(B)** Air pressure measurements of chamber 1. **(C)** Lumped disturbance estimation. Yellow, red and blue indicate the tracking, compliance, and recovery modes. Green indicates the autonomous switching between the compliance and recovery modes.

Experimental results for the second scenario are shown in Figures 6, 7. The position tracking results for the SMC and SMART approaches are depicted in Figures 6B, Figure 7A, respectively. Note that both controllers can track the original trajectory after the obstacle is moved out of the way. However, the SMC approach shows a significant overshoot after the object is moved away. The high chamber pressure during contact contributes to this overshoot problem. As presented in Figure 6C, the SMC approach saturates the fabric actuator during the contact. On the other hand, the SMART controller maintains the low chamber pressure during the contact, as shown in Figure 6B. The low chamber pressure also indicates that the actuator is compliant with the obstacle. It is also observed that the disturbance estimation changes with a different desired trajectory in each mode, as shown in Figure 7C.

The performance of the controllers for contacting with a stiff obstacle is evaluated through two metrics: the normalized chamber air pressure (NCAP) during the contact and the absolute value of maximum position overshoot (MPO) immediately after the contact. The NCAP reflects the level of compliance during contact, and the chamber pressure is normalized with respect to the maximum air pressure allowed for the actuator. The MPO reflects the position tracking performance after the contact. A smaller value of MPO indicates higher precision in the goal-reaching task. The metrics are computed over three trials, and the NCAP values for SMC and SMART controllers are 0.9293 and 0.7538, while the MPO (rad) values are 0.1511 and 0.0427, respectively. It is observed that the SMART controller outperforms the conventional SMC in both criteria.

**FIGURE 8:**
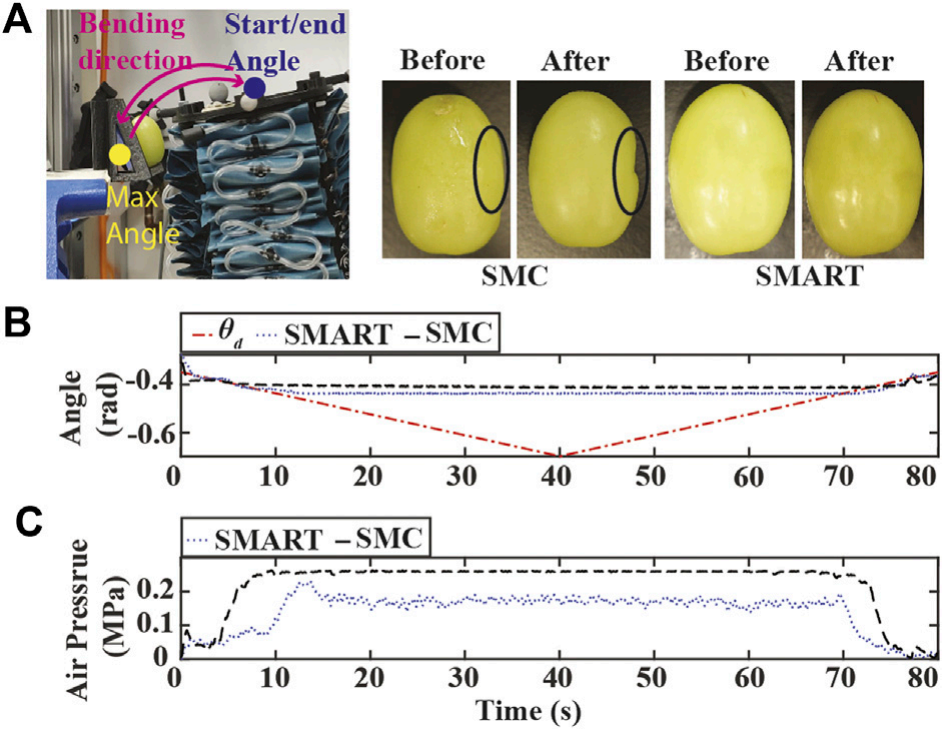
Experiment results of the SMART approach tracking bending angle with compliant obstacle. **(A)** Experiment setup and images of green grapes. **(B)** Bending angle tracking results where θ_d_ is the original desired bending angles (dash-dotted red) **(C)** Air pressure measurements of chamber 1.

The experimental results for the third scenario are in Figure 8. As presented in Figure 8A, the soft robot with the conventional SMC approach crushes into the grape. On the other hand, both controllers can track the set-point when it is far away from the obstacle, as seen in Figure 8B. Similar to the stiff obstacle case, the SMART controller can maintain low air pressure during contact, as presented in Figure 8C. A video demonstrating all three scenarios is available at https://youtu.be/3QqwjNVIfeo.

### 5.3 Evaluation of SMART design parameters

**FIGURE 9:**
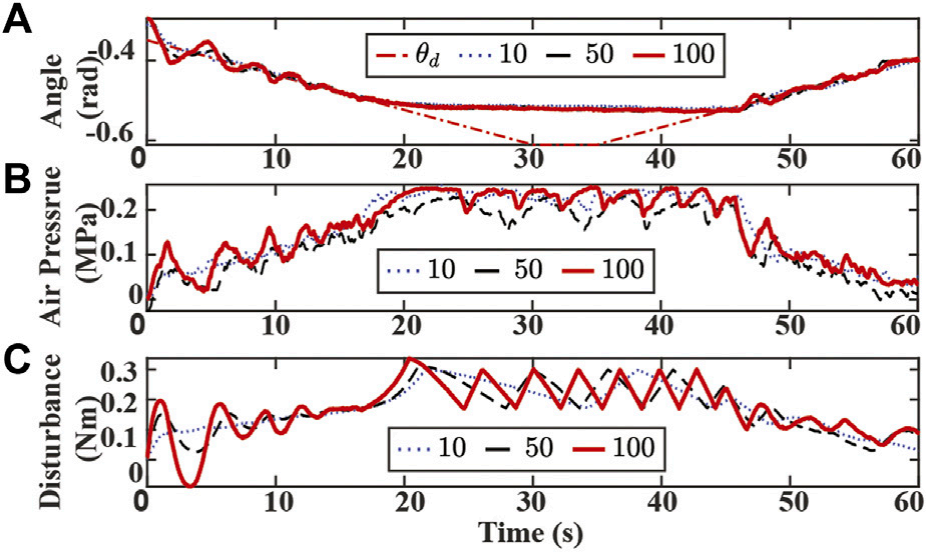
Experiment results of tracking bending angle with obstacle and changing k_sf_. **(A)** Bending angle tracking results where θ is the original desired (dash-dotted red), and k_sf_ are set to 10 (dotted blue), 50 (dashed black), 100 (solid red), respectively. **(B)** Air pressure measurements of chamber 1. **(C)** Lumped disturbance estimation.

Experimental results with varying k_sf_ are presented in Figure 9. A larger k_sf_ value results in a higher oscillation in bending angle tracking, chamber air pressures, and estimated disturbance at the beginning and end of the experiments. Since the switching algorithm utilizes tracking error and estimated disturbance value to detect possible contacts, the oscillation could lead to false contact detection during the tracking or recovery mode. On the other hand, the SMART controller is more sensitive to the possible contact with larger k_sf_. As shown in Figure 9C, the estimated disturbance grows faster with the increase of k_sf_ when the actuator is initially in contact with the obstacle around 20 s. While the desired bending angle varies in different modes, the controller with a smaller k_sf_ converges slower to the new desired angle, which is depicted in 20–40 s time interval in Figure 9C. The slow convergence speed could make the controller unaware of the detachment between the obstacle and the actuator.

**FIGURE 10:**
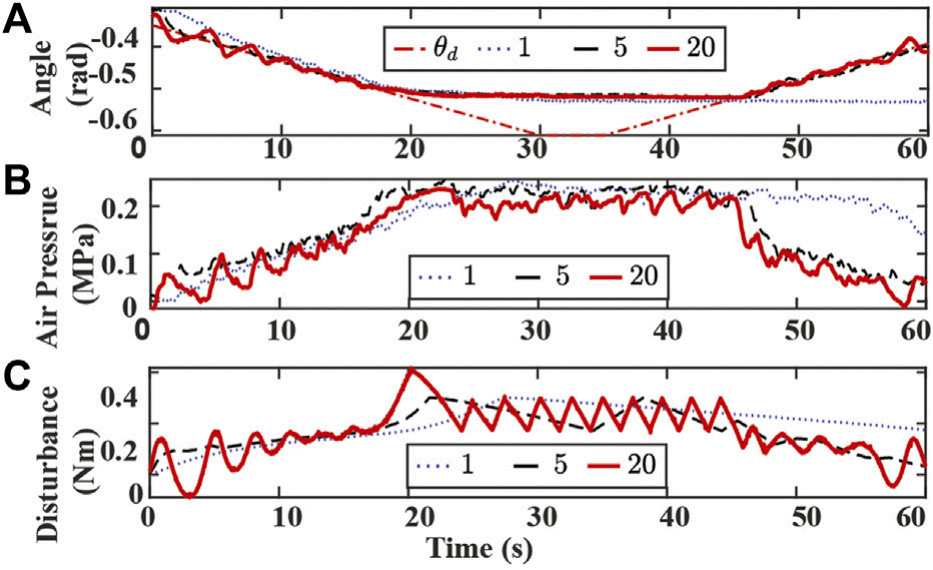
Experiment results of tracking bending angle with obstacle and changing k_w_. **(A)** Bending angle tracking results where θ is the original desired (dash-dotted red), and k_w_ are set to 1 (dotted blue), 5 (dashed black), 20 (solid red), respectively. **(B)** Air pressure measurements of chamber 1. **(C)** Lumped disturbance estimation.

Experimental results with varying k_w_ are presented in Figure 10. A small k_w_ value requires a longer time to converge to the new desired trajectory and fails to track the bending angle reference at the end of the experiment, as shown in Figure 10A. Similar to the k_sf_ case, a larger k_w_ value results in a more significant oscillation in bending angle tracking, chamber air pressures, and estimated disturbance at the beginning and the end of the experiment. Although the SMART controller with a large k_w_ value is more sensitive to the contact event, a higher peak value of disturbance estimation is also observed at 20 s, and a more dense zigzag pattern is also observed between 20 to 50 s, as shown in Figure 10C.

**FIGURE 11:**
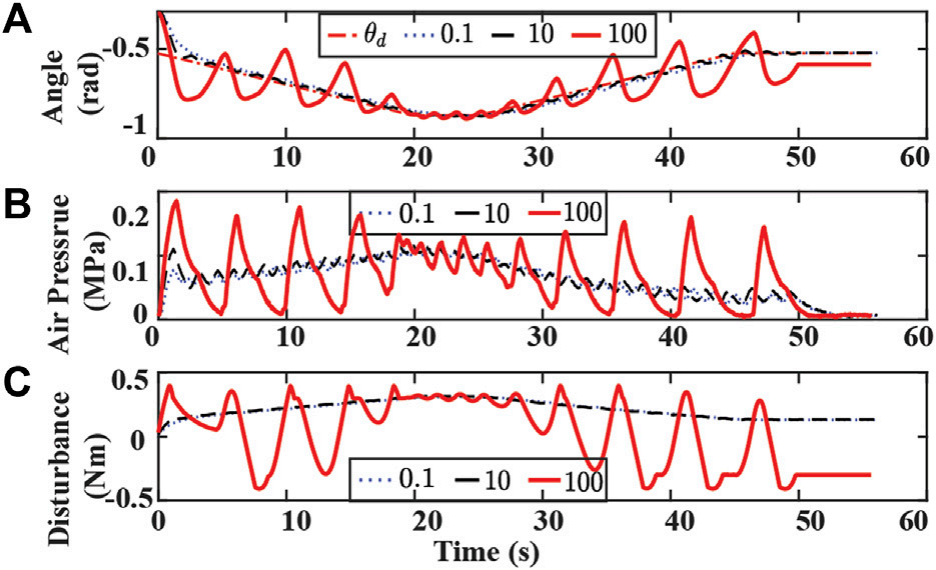
Experiment results of tracking bending angle without obstacle and changing η. **(A)** Bending angle tracking results where θ is the original desired (dash-dotted red), and η are set to 0.1 (dotted blue), 10 (dashed black), 100 (solid red), respectively. **(B)** Air pressure measurements of chamber 1. **(C)** Lumped disturbance estimation.

**FIGURE 12:**
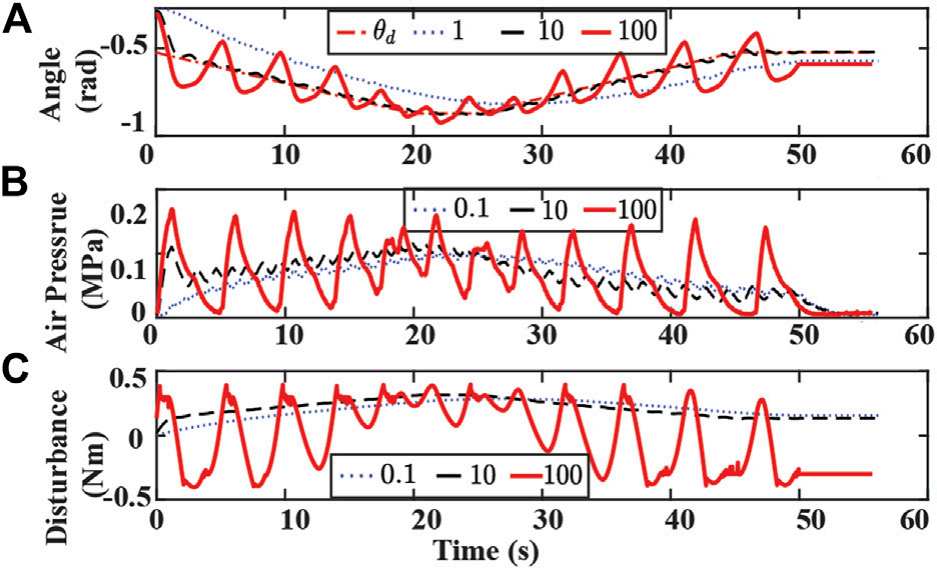
Experiment results of tracking bending angle with obstacle and changing λ. **(A)** Bending angle tracking results where θ is the original desired (dash-dotted red), and λ are set to 0.1 (dotted blue), 10 (dashed black), 100 (solid red), respectively. **(B)** Air pressure measurements of chamber 1. **(C)** Lumped disturbance estimation.

Experimental results with varying η and λ are presented in Figures 11, 12,respectively. Controller with a small η value uses a longer time to reach the desired trajectory. The controller with small λ fails to track the desired trajectory. A large value of η or λ results in significant oscillation in the bending angle tracking and false contact detection when no obstacle is on the path.

## 6 Conclusion

This paper presents a SMART controller for a fabric-based soft actuator. The controller aims to autonomously adjust the actuator’s desired trajectory and maintain compliance with the obstacle during a goal reaching task. A new model parameter was introduced to reflect the distance between the center line and the inextensible arc to improve the model accuracy. The proposed controller contains three main parts: an NDOB, a three-mode switching algorithm, and an SMC approach. The NDOB was designed to estimate the lumped disturbance, which included modeling uncertainties and external load. The three-mode switching algorithm was integrated with the baseline SMC and NDOB to detect contacts and adjust the actuator’s desired trajectory. Experimental results indicated that the SMART controller was more compliant during the contact and more precise in the goal-reaching task after the contact than the baseline SMC. The convergence speed of the NDOB in the SMART controller increases with a larger k_sf_ and k_w_ value with a cost of oscillations in position tracking. A small k_sf_ could miss the detachment between the actuator and the obstacle, and a small k_w_ value failed to track the set-point. The controller with a small value of η or λ requires a longer time to reach the desired path or even fails to track the path. A large η or λ value results in false contact detection and generates significant oscillation in the bending angle tracking.

Future studies include optimizing the design parameter to minimize the total control energy, and implementing other switching logic for autonomously detaching from the obstacle and re-planning the trajectory. An extended dynamic model which includes the current model and the model of the low-level pressure dynamics will also be studied to improve the performance of the proposed controller in more dynamic tasks. We will also expand the dynamic model and SMART controller to the two bending angles of one actuator and a soft arm with three serially connected actuators for more complex tasks. In addition, we are interested in incorporating embodied kinematic sensors such as wire encoders to estimate the lumped disturbance and exploring the intelligent interaction controller between the soft arm and other objects, such as human users.

We will also expand the dynamic model and SMART controller to the two bending angles of one actuator and a soft arm with three serially connected actuators for more complex tasks. In addition, we are interested in incorporating embodied kinematic sensors such as wire encoders to estimate the lumped disturbance and exploring the intelligent interaction controller between the soft arm and other objects, such as human users.

## Data Availability

The raw data supporting the conclusion of this article will be made available by the authors, without undue reservation.
